# Epidemiological indicators of accidental laboratory-origin outbreaks

**DOI:** 10.1017/S0950268825100915

**Published:** 2026-01-02

**Authors:** Sandhya Dhawan, Wirichada Pan-ngum, Chandini Raina MacIntyre, Stuart D. Blacksell

**Affiliations:** 1 Mahidol Oxford Tropical Research Medicine Unit, Thailand; 2 Kirby Institute, University of New South Wales, Australia; 3 University of Oxford, Oxford, UK

**Keywords:** accidental pathogen release, laboratory leaks, laboratory outbreaks, pathogen escapes, unnatural epidemic events

## Abstract

Accidental escapes of pathogens from laboratories continue to cause outbreaks in the community today, posing significant risks to the general public, animal communities and the environment. These incidents, as well as the uncertainties surrounding the origins of the COVID-19 pandemic, highlight the need to consider unnatural origins as part of emerging outbreak surveillance and detection. Identifying recurring patterns and distinctive factors of laboratory-associated disease outbreaks can aid in successfully preventing and mitigating these occurrences. Seventy incidents of laboratory-associated leaks that led to outbreaks in the wider public have been reported (Supplementary Appendix S1). Seven renowned cases that have been comprehensively studied were selected for review: (i) 1955 Polio vaccine incident in western USA, (ii) 1977 H1N1 influenza virus re-emergence in China and the Soviet Union, (iii) 1979 Anthrax release in Sverdlovsk, Soviet Union, (iv) 1995 Venezuelan equine encephalitis epidemics in Venezuela and Colombia, (v) 2003–4 SARS-CoV-1 escapes from Singapore, Taiwan and China, (vi) 2007 Foot-and-Mouth disease virus outbreak in Pirbright, England and (vii) 2019 Brucella leak in Lanzhou, China. These outbreaks were selected because data on their geographical spread, genetics, phylogeny, epidemiological factors (including attack rates, infectious dose, time, location and season of spread) and governmental and institutional responses to the incidents had been previously analysed and published. Thematic analysis of these lines of evidence revealed seven recurring insights described in historically confirmed laboratory-associated outbreaks: unusual strain characteristics, peculiar clinical manifestations or affected demographics, unusual geographical features, atypical epidemiological patterns, delayed government action and communication to the public, misinformation and disinformation spread to the public and biosafety concerns/incidents predating the event. The outbreaks exhibited between 13 and 19 retrospectively identified indicators. These indicators were used to develop preliminary risk criteria intended to support structured, hypothesis-generating assessment of outbreaks, rather than to establish origin.

## Introduction

Despite continual advances in biosafety and biosecurity policies, accidental pathogen escapes from laboratories continue to cause disease outbreaks in the community. The question is not if a pathogen will escape, but rather which pathogen will and what measures are in place to contain an escape with serious consequences [1]. Past laboratory-origin epidemics [2–4] and outbreaks of unknown origin [5], namely the Severe Acute Respiratory Syndrome Coronavirus 2 (SARS-CoV-2) pandemic (2019–2023) [6], underscore the need to consider unnatural origins when identifying outbreaks.

Different lines of evidence, including phylogenetics, epidemiology, seroepidemiology and criminal or geopolitical intelligence, are required to determine whether an outbreak is of unnatural origin [7]. Phylogenetics alone may not identify pathogens of laboratory origin because serial passaging a pathogen through an animal host will produce genetic markers that appear to be of natural origin [7]. To investigate distinctive factors of laboratory-origin outbreaks, historically confirmed incidents should be studied to identify emerging themes and indicators.

A total of 70 incidents of accidental laboratory leaks have been reported, with the earliest recorded in 1901 and the most recent in 2024 (Appendix, Supplementary Table 1). Of these incidents, 56 (80.0%) resulted in community cases and 29 (41.4%) resulted in fatalities. Seven well-known cases that have bee comprehensively studied were selected for review: (i) 1955 Polio vaccine incident in western USA, (ii) 1977 H1N1 influenza virus re-emegence in China and the Soviet Union, (iii) 1979 Anthrax release in Sverdlovsk, Soviet Union, (iv) 1995 Venezuelan Equine Encephalitis epidemics in Venezuela and Colombia, (v) 2003-4 SARS-CoV-1 escapes from Singapore, Taiwan, and China, (vi) 2007 Foot-and-Mouth disease virus outbreak in Pirbright, England, and (vii) 2019 Brucella leak in Lanzhou, China, were comprehensively evaluated for insights into their geographical, epidemiological, phylogenetic and other characteristics. These indicators were used to explore whether similar epidemiological features have been discussed in relation to the SARS-CoV-2 pandemic, without implying causality.

## Methods

A literature review was conducted across the PubMed, ProMED-Mail, Scopus and Web of Science databases using keywords to identify published literature on the seven laboratory-confirmed outbreaks. Public information accessible on the World Health Organization (WHO) and the Centres for Disease Control (CDC) platforms was gathered, as well as relevant news articles, government reports, correspondence and grey literature published during the respective outbreaks. The information collected included historical facts, witness accounts, outbreak investigations, characteristics of the outbreak and strain, epidemiological parameters and descriptive statistics. An article or report was excluded if it contained no information on any risk variables analysed (geographical spread, genetics and phylogeny, epidemiological factors, timely and accurate reporting/infodemic).

## Summary of case studies

### The Cutter Laboratories polio vaccine trials across the western United States

Poliomyelitis epidemics plagued the world in the 1950s, leading to intensive research into the development of inactivated or live-attenuated vaccines for poliovirus [8]. In April 1955, Cutter Laboratories in California was licenced to produce the Salk formaldehyde-inactivated polio vaccine (IPV), following successful trials [9]. However, some batches produced by Cutter Laboratories were insufficiently inactivated and contained live poliovirus [10, 11]. Multiple children received these contaminated doses, leading to tens of thousands of abortive infections, dozens of paralytic cases and several deaths, including secondary transmission within families and communities [11, 12]. The incident shook public trust in vaccines, reshaped vaccine policy and became a defining moment in the history of vaccine safety.

#### Geographical spread

Approximately 120,000 contaminated doses were administered to primarily grade-school children, and roughly 400,000 people received the Cutter vaccine during a 10-day period in mid-April. A majority of them developed abortive polio [8]. Most cases occurred between late April and May 1955, then declined sharply by June, aligning with the vaccination window [11]. Ultimately, at least 220,000 people were exposed, including 100,000 household contacts of immunized children, resulting in 164 cases of severe paralysis and 10 deaths [11].

Infections clustered in states where the Cutter vaccine was widely used. California and Idaho experienced the highest numbers, while nearby states saw smaller outbreaks. Idaho typically reported very low polio incidence (11 cases) during April–June in 1950 to 1954. In 1955, however, an eightfold increase (88 cases) was observed, of which 84 were attributable to vaccine–associated cases [13].

#### Genetic and clinical evidence

Paralytic poliomyelitis developed 4 to 10 days after vaccination in patients [11], with paralysis typically beginning in the inoculated arm, a pattern less common in natural poliomyelitis cases [12, 13]. All early cases were linked to Cutter vaccine recipients, with secondary cases in their families and community contacts [13, 14]. Severe neurological complications involving the central nervous system were noted in those who were administered the vaccine, as compared to family contacts as well [14]. The incidence of paralytic disease peaked in children aged 7 across the western US, reflecting an unusually high concentration of poliomyelitis in children, likely because they were heavily vaccinated under school programs in this region [15].

Laboratory investigations revealed that live type 1 poliovirus (the causative agent of the Cutter vaccine-associated cases) was isolated from 7 of 8 vaccine lots, demonstrating failed inactivation. Additional findings of types 2 and 3 poliovirus in certain lots underscored multiple strains of poliovirus circulating at the time, even though types 2 and 3 did not result in clinical cases [14, 16].

#### Epidemiological factors

The number of cases among Cutter vaccine recipients and their contacts far exceeded what would be expected for natural poliomyelitis at the time, and vaccine recipients from other manufacturers showed no such pattern [13]. Moreover, Cutter vaccine-associated cases declined as cases of seasonal poliomyelitis began to increase [13].

Incubation periods among vaccinated children ranged from 4 to 15 days, shorter than the typical interval for naturally occurring polio, whereas contact cases showed incubation periods consistent with secondary transmission [11]. Vaccinated cases peaked approximately 1 week after vaccination, and secondary cases peaked 3 weeks after the midpoint of vaccination. The appearance of cases in waves is suggestive of a common-source outbreak [13].

Attack rate analysis of the vaccine lots revealed that two of three production pools were inadequately inactivated and accounted for a more than 10-fold increase in paralytic cases [11]. Secondary attack rates in household contacts were similar to those seen in natural poliomyelitis, with limited spread to wider community contacts [13].

#### Timely and accurate reporting/Infodemic

The Salk vaccine was licenced within a day under political pressure and distributed widely within two weeks [10]. Although the vaccine had passed required safety testing [16], biosafety concerns at Cutter Laboratories were identified, including the use of the highly virulent Mahoney strain, insufficient viral inactivation and inadequate safety testing [10]. The vaccine was swiftly withdrawn on April 27 after cases rose sharply [11].

Overall, communication with other scientists and the government was poor. Swedish researchers, such as Sven Gard, presented research showing that Salk’s vaccine inactivation procedure was ineffective [17–19]. Despite these concerns, Salk did not make the proposed changes, and the vaccine trial was launched in 1954, even though regulators lacked the capacity to validate each dose during production, relying on manufacturers for quality assessment [10, 17].

In fact, a similar incident was documented in which another company using the Salk IPV, Wyeth Laboratories, was responsible for 37 vaccine-associated poliomyelitis cases. Yet, the report was kept confidential from public health authorities and the public [10, 17].

Media further contributed to misinformation and infodemics, which eroded public trust in vaccines. Vaccine rates significantly dropped across the world [17, 20]. The Cutter polio vaccine incident contributed to the shift toward Sabin’s oral polio vaccine (OPV) in the 1960s [8]. Both the Salk and Sabin vaccines were also found to have been contaminated with Simian Vacuolating Virus (SV40) due to inadequate formaldehyde inactivation of the monkey kidney cells used to cultivate poliovirus [21]. This led to SV40-contaminated polio vaccines being administered to millions of people between 1955 and 1963 [8].

**Table 1.** Summary details of selected case studies of laboratory outbreaks

Year	Location	Pathogen	Cases	Exposures	Fatalities (reported)	Outbreak details
1955	United States	Poliovirus	40,000	Unknown	10	A batch of Salk’s inactivated poliovirus vaccine (IPV), manufactured at Cutter Laboratories, was inadequately inactivated with formaldehyde during production. About 120,000 doses were administered, and led to ˜40,000 children contracting polio. At least 220,000 people were exposed to live poliovirus via the vaccines, including 100,000 household contacts of immunized children, with 10 deaths recorded.
1977	China / Soviet Union	Influenza A H1N1 virus	21	Unknown	Not reliably quantified	A novel H1N1 influenza virus re-emerged in northeast China and Soviet Union in 1977, likely caused by an accidental release incident from a Chinese or Russian vaccine facility cultivating a c1950 H1N1 virus in response to the US ‘swine flu’ program launched in the aftermath of the Fort Dix outbreak.
1979	Soviet Union	*Bacillus anthracis*	96	Unknown	96 or 105	Four virulent strains of *B. anthracis* were released and circulating in Sverdlovsk and a nearby town in Soviet Union in 1979, due to oversight of a missing filter in the exhaust vent of the Anthrax production facility in Compound 19.
1995	Venezuela, Colombia	Venezuelan equine encephalitis virus (VEE)	≥100,000	Unknown	~300	Multiple VEE outbreaks arose from either an infected laboratory worker, or escape and aerosolization of the 1938–1973 strain traced back to a virology lab in Venezuela via clinical analysis of patient samples. The epidemic resulted in over 100,000 infected and 300 dead.
2003	Singapore	Severe Acute Respiratory Syndrome Coronavirus (SARS-CoV-1)	1	84	0	A graduate student in Singapore contracted SARS in a BSL–3 laboratory at the Institute of Environmental Health (EHI) while handling West Nile Virus samples that had been cross-contaminated with a SARS-CoV isolate (Figure 1). He further exposed 8 household, 32 work and 2 community contacts.
2003	Taiwan	SARS-CoV-1	1	95	0	A research scientist at the Taiwan Military Institute of Preventative Medical Research (IPMR) contracted a SARS-CoV infection due to inadequate decontamination and disposal of infectious waste. The research scientist travelled to a conference while ill, exposing fellow passengers and airline staff (Figure 1).
2004	China	SARS-CoV-1	11	747	1	Two doctoral research students working at the Chinese National Institute of Virology (NIV) acquired a SARS-CoV infection from cross-contamination in, likely, the electron microscopy room. One of the students transmitted the infection to her mother (who died as a consequence of taking care of her) and her attending nurse. The nurse also transferred her illness to three of her relatives, a co-patient and their relative (Figure 2). Serological staff surveillance of laboratory workers at NIV revealed two more graduate students from the same department who contracted the virus due to improper inactivation of a SARS-CoV sample two months prior.
2007	United Kingdom	Foot-and-Mouth Disease virus (FMDV)	278	Unknown	1,578 culled	A c1967 FMDV strain leaked from a damaged waste-water pipe at the Pirbright campus, likely released from the BSL3 laboratory at the Institute for Animal Health or Merial vaccine plant due to improper attenuation. The virus spread to eight farms and incurred a £200 million loss to the UK economy.
2019	China	*Brucella abortus* A19	10,528	68,571	0	The use of expired disinfectants led to Brucella-contaminated waste gas being leaked from fermentation tanks at the Zhongmu Lanzhou Biopharmaceutical Plant. Aerosols spread to the neighbouring institute and communities.

*Note:* Fatality figures reflect officially reported deaths at the time of investigation; where reliable global estimates are unavailable or contested, entries are qualified to indicate uncertainty or under-reporting. “Unknown” indicates that exposure counts could not be reliably reconstructed from available contemporaneous records or retrospective analyses.

### The re-emergence of the H1N1 influenza virus in China and the Soviet Union

Every pandemic influenza strain has replaced its predecessor strain [22, 23]. However, in 1977, two serotype A viruses were recorded to co-circulate for the first time in history: the dominant H3N2 subtype and the previously extinct human influenza A H1N1 virus [24]. This situation was due to an accidental release during laboratory activities.

#### Geographical spread

The H1N1 influenza re-emerged in northeast China in May 1977 and soon after in the eastern Soviet Union [25]. The Soviet Union reported the outbreak to the WHO in September 1977, followed by Chinese reports in May 1978 [26]. The c.1957 H1N1 virus strain initially spread in the Soviet Union, Hong Kong and China, then rapidly worldwide, causing mild infections in individuals under 21 while excess mortality was largely confined to older populations, with global death estimates varying widely. [26, 27].

#### Genetic and clinical evidence

Genetic analysis showed that the 1977 H1N1 virus outbreak strain was closely related to strains from 1949–1950 but distinct from the 1947 or 1957 strain [27], suggesting it had likely been preserved since 1950 [27] and accidentally released when population immunity to H1 and N1 antigens declined [8].

Many isolates from the outbreak were temperature-sensitive, a marker of laboratory manipulation distinctive to live attenuated influenza vaccine (LAIV) studies. However, not all strains were temperature-sensitive [28]; a mixed population of strains suggests a possible escape event during the temperature-sensitivity selection experiment [26, 28]. The outbreak strain had low virulence, varied attack rates within the same region and a low mortality rate, likely owing to attenuation and pre-existing immunity in the older population [1, 26].

#### Epidemiological factors

The H1N1 virus spread more slowly nationwide in Liaoning (May–October) than the Asian H2N2 pandemic in 1957 (February to March) [26]. This unusual disease progression may have been due to an unfavourable season, although off-season outbreaks suggest an unnatural origin.

Two factors suggest that an incompletely attenuated vaccine strain caused the outbreak: ongoing research on LAIV at the time [1] and the renewed interest in prophylaxis following the 1976 H1N1 outbreak at Fort Dix, New Jersey [29]. It is plausible that a Chinese or Russian vaccine facility thawed and cultivated a c.1950 H1N1 influenza virus in response to the US ‘swine flu’ program launched in the aftermath of the Fort Dix outbreak [1].

#### Timely and accurate reporting/Infodemic

The source was debated, with suggestions of an accidental escape being refuted by Chinese and Soviet virologists [26]. Western scientists refrained from discussing the laboratory-origin theory to foster collaboration amid Cold War tension [1]. Natural-origin hypotheses included the possibility of viral latency in an unspecified animal reservoir. In 2006, a paper suggested that the virus emerged from migratory birds at Siberian lakes, after isolating strains mistaken for avian H1N1 influenza virus from meltwater. The paper was criticised in 2008, where direct evidence demonstrated that the meltwater strain, ironically, was also leaked from a laboratory [30]. In 2009–2010, the laboratory release theory became widely accepted [1].

### The release of inhalational anthrax from an exhaust vent in Sverdlovsk, Soviet Union

After WWII, the Soviet Union established an anthrax production plant [31] in their Military Research Facility: Compound 19 in Sverdlovsk. The causative agent, *Bacillus anthracis*, mainly affects domestic animals and, occasionally, humans via cutaneous transfer or, rarely, through ingestion or inhalation [32]. Natural anthrax is almost always cutaneous, and inhalational anthrax should raise suspicion towards a deliberate event [32].

A clogged filter in the exhaust vent was removed but not replaced; machines ran for several hours as usual [31]. Anthrax aerosols escaped to a ceramic plant and a town nearby, where many workers were discovered ill. Within a week, most exposed workers had died, and hospitals received an influx of patients from different towns [31].

#### Geographical spread

According to Soviet reports, the epidemic began in late March, took place from 4^th^ April to 18^th^ May 1979, and caused a total of 96 cases with 66 fatalities [2]. Witnesses claimed a death toll of ~105 [31], and an article quoted as many as a thousand deaths [32]. The actual number of human fatalities or cases remains unknown, as it was reported that the KGB destroyed most hospital records [31, 33]. In an attempt to conceal the truth, the incident was falsely attributed to gastrointestinal anthrax (a rare manifestation) resulting from consumption of anthrax-contaminated meat [31].

#### Genetic and clinical evidence

Genetic studies dated the accident to April 3^rd^ or 4^th,^ 1979, consistent with the observed anthrax incubation period. The Soviet officials falsely reported the start date as March 30^th^ 1979, manipulated medical records of early cases and issued fabricated death reports to the victims’ families as part of the cover-up [31]. They also denied inhalational anthrax, although this was later confirmed from autopsy data [33].

#### Epidemiological factors

It was estimated that victims were exposed to a far lower infectious dose (~1–10 or 100–2,000 spores) than observed for naturally occurring inhalational anthrax (8,000–10,000 spores) [34], signifying a potentially weaponised strain. This is consistent with early clinical studies, where more than four virulent strains of *B. anthracis* circulating during the accident were all traced to the biological weapons facility [35].

Most infected patients worked or lived close to the military facility (within 4 km)^35^, with animal cases detected up to 60 km away [2]. The aerosol size was estimated to be <5–10 μm to have allowed for extended dispersal and prolonged infection, more extensive than that observed in the 2001 ‘Amerithrax’ attacks (<5 μm) [34].

Autopsies confirmed that fatal cases resulted from inhalation exposure [33], a rare clinical form of natural anthrax [34]. The mean incubation period of the Sverdlovsk accident (~10 days, with some cases appearing after 43 days)^2^ was longer than that of naturally occurring anthrax outbreaks and even the 2001 Amerithrax intentional anthrax release (4–6 days)^35^.

#### Timely and accurate reporting/infodemic

In November 1979, a Russian magazine reported that in April, ‘an explosion in the military facility of Sverdlovsk had released a cloud of deadly bacteria’ [31]. Western agencies later picked up coverage of the outbreak [2], alleging it was a violation of the 1972 Biological Weapons Convention, although all claims of a laboratory accident were denied.

Workers in Compound 19 raised biosafety concerns about airborne spores in the laboratory, clogged filters and neglected maintenance checks [31]. After the accident, senior officers were alerted, but city officials and the Ministry of Defence in Moscow were not informed.

On June 12, 1980, residents of Sverdlovsk were informed that the outbreak was caused by contaminated meat from illegal wet market stalls, leading to the culling of more than 100 stray dogs and animals in the vicinity [31].

Soviet authorities denied requests to permit independent scientists to investigate the incident [2]. An ‘information war’ arose between those doubting a natural outbreak and those convinced of its natural origin. It took nine years for Soviet medical experts to disclose information about the Sverdlovsk incident to the US and thirteen years for then-Soviet President Boris Yeltsin to admit to the accident [1].

### The VEE epidemics in Venezuela and Colombia

In 1995, one of the largest epidemics of VEE was documented in Venezuela and Colombia [8]. VEE is an arboviral disease transmitted by mosquitoes that causes intermittent epizootics and sometimes human epidemics across the Americas. Equine disease is severe, while human infections range from asymptomatic to acute febrile illness with neurological complications, and fatality rates of 4 to 14% [1, 8]. Naturally, VEE circulates at low levels in enzootic cycles. The enzootic strains (ID, IE, IF, II–VI) rarely cause major outbreaks, which occur only when an enzootic strain mutates into an epizootic subtype (IAB or IC) that efficiently amplifies in equines and drives widespread human spillover [36, 37].

**Figure 1.**
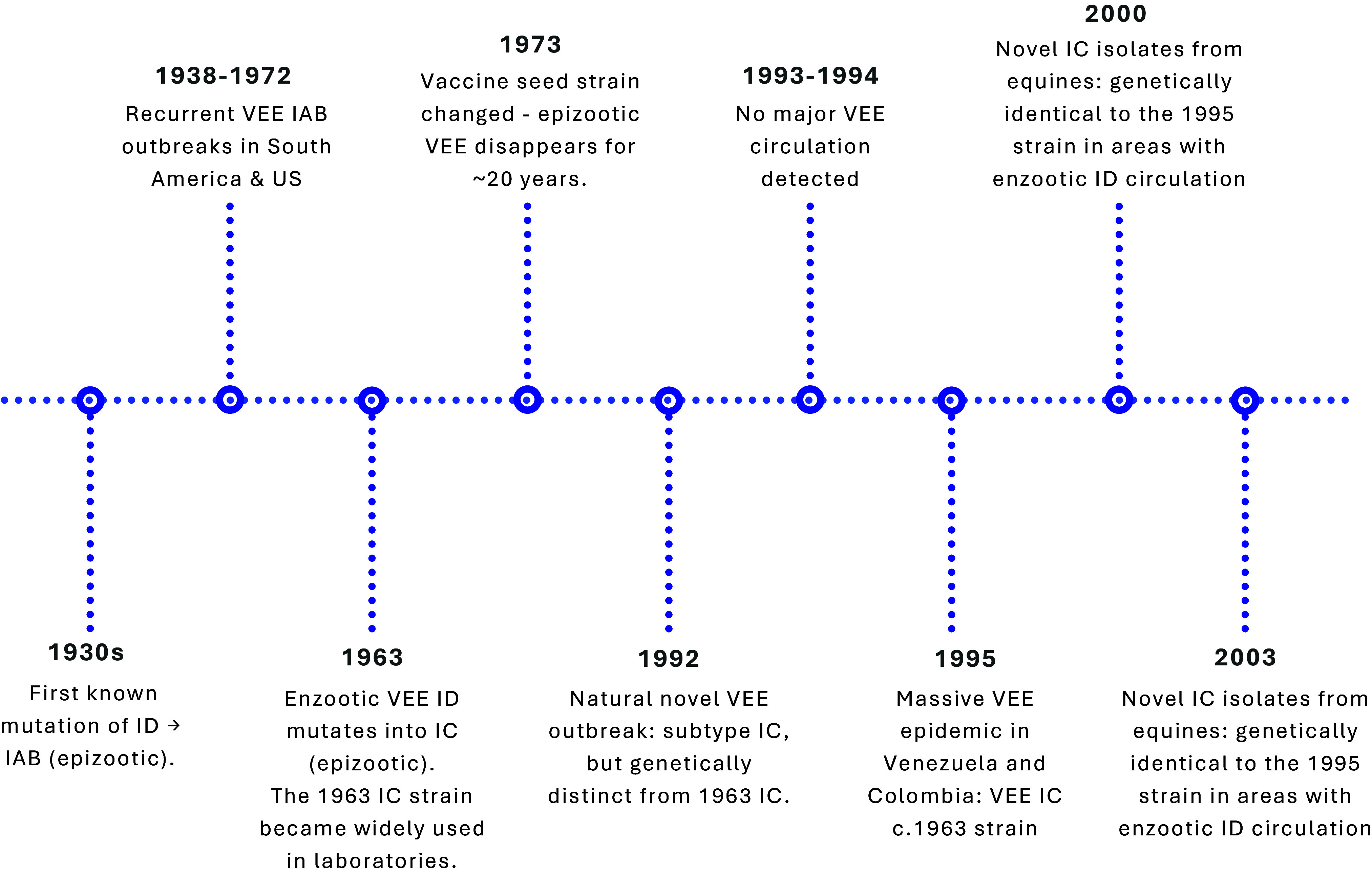
Timeline of Venezuelan equine encephalitis (VEE) virus outbreaks and laboratory-associated events.

Epizootic strains have mutated from enzootic only three times (ID→IAB in the 1930s; ID→IC in 1963 and again in 1992). However, many VEE outbreaks reported from the late 1930s through early 1970s were traced to inadequately inactivated veterinary vaccines derived from the 1938 IAB strain [38, 39]. Residual live virus in the vaccines repeatedly sparked outbreaks until the seed strain was replaced with an attenuated variant in 1973, after which epizootics ceased for nearly 20 years [38, 40] (Figure 1). Unlike the IAB strains, there is no record that subtype IC strains were ever used in vaccine production, hence an unlikely source of the 1995 outbreak.

The 1995 outbreak in Venezuela and Colombia was unusual because this strain matched an IC virus used in diagnostic reagents in a local virology laboratory, one previously shown to contain live virus. Many investigators concluded that the 1995 epidemic most likely resulted from an inadvertent laboratory escape rather than natural evolutionary emergence [41, 42].

#### Geographical spread

In April 1995, veterinarians in Venezuela first detected equine deaths suggestive of VEE, followed by human febrile cases [43]. The outbreak began in eastern Falcón State, Venezuela and spread westward across states by mid-July. Transmission intensified in rural areas by late August, and by September–October, large numbers of human and equine cases were reported in the Colombian state of La Guajira [42, 44]. Overall, the epidemic caused ≥ 100,000 human cases and ~ 300 deaths [41–43].

VEE outbreaks typically emerge in regions with known enzootic subtype ID circulation, and within localized equine–mosquito amplification cycles [36, 37]. The only historical exception was the 1969–1971 outbreak originating in the Guajira peninsula, although the area had prior enzootic ID activity [45]. In contrast, the 1995 outbreak began abruptly in Falcón State, a region with no record of circulation of closely related enzootic ID progenitor strains [46], or of laboratories or vaccine production facilities in close proximity [47].

Heavy rainfall in the normally arid Guajira region increased vector densities and expanded the spread [42]. However, unlike natural transmission patterns, the outbreak spread rapidly through rural areas with limited equine populations, suggesting that human–mosquito–human transmission was also occurring.

Importantly, the 1995 virus was identical to a subtype IC antigen strain that was in regular use for antigen preparation in laboratories near the outbreak area at the time [41].

#### Genetic and clinical evidence

Genomic analyses showed that the 1995 outbreak virus was subtype IC, which had previously caused two other major epidemics (1962 to 1963 and 1992 to 1993). It was a genetic match to a strain isolated in 1963, which had disappeared from nature 30 years ago [41]. The 1995 viral sequence showed almost no evolutionary change during the interepidemic period, inconsistent with estimates of epidemic and enzootic VEE virus evolution rates, which indicate a relatively steady rate of nucleotide substitutions, on the order of 2–4 × 10^−4^ substitutions/nucleotide/year [48–51].

Phylogenetic analysis demonstrated sequence identity to the P676-ag virus, isolated from a 1982 antigen preparation used for diagnostic testing in Venezuela [41], explaining the genetic conservation between the epidemic events.

Clinically, infected humans exhibited high viremias comparable to those in equines, sufficient to infect the epidemic mosquito vector [42, 52, 53]. Higher disease incidence was observed in unimmunized equines, particularly donkeys, due to low equine immunization rates in the region [43].

#### Epidemiological factors

The 1995 VEE epidemic displayed attack rates of ~ 36%, with some communities reporting rates as high as ~ 93%, far exceeding typical VEE epizootic patterns [43]. However, secondary attack rates were low in Colombian communities, and no secondary infections occurred among Venezuelan healthcare workers, indicating low person-to-person transmission despite extensive community spread [54].

Field studies, before and after the epidemic, found no evidence of ongoing circulation of epizootic IAB or IC strains [45, 46, 55], or enzootic ID viruses genetically related to the 1995 IC strains in northern Venezuela, demonstrating an absence of local natural reservoirs for the disease [46].

Natural transmission often generates genetic shift or drift due to low-dose mosquito transmission, which was not observed with the implicated strain [56, 57]. Furthermore, the much faster geographic spread of the 1995 outbreak compared to the natural 1962–1964 epizootic suggests an atypical introduction rather than gradual local emergence [42].

#### Timely and accurate reporting/Infodemic

The Colombian Ministry of Health and Animal Health Service deployed timely surveillance across La Guajira, generating real-time intelligence to guide vector control, equine vaccination and movement restrictions. Implementation of early interventions, in line with animal health regulations, helped prevent wider domestic or international spread at a time when heavy rainfall had increased vector density and heightened epidemic risk [44].

While the spread of infodemic during the outbreak was limited, scientific discourse on the origins of the virus emerged years after the epidemic [41]. Between 2000 and 2003, outbreaks of a subtype IC strain genetically identical to the 1995 virus were reported in Venezuela, despite the strain no longer being widely used in laboratories (Figure 1) [47]. From 1995 to 2000, this IC lineage showed a ≈ 10-fold slower evolutionary rate, implying limited replication compared with typical mammal, mosquito, or equine transmission cycles [47]. Field investigations failed to identify reservoir hosts or vectors, and the outbreaks occurred at the end of the rainy season, which is not typical of the VEE epidemic pattern [47]. The 2000 strains also did not cluster phylogenetically with the P676-ag strain, and the 2000s outbreak locations were not near any diagnostic or vaccine production laboratories that work with VEE virus either [47]. These findings suggest that the 2000 outbreaks involved naturally circulating strains that have remained genetically stable since the 1995 laboratory release. While these anomalies led the VEE working group to reconsider their suggestion of a laboratory origin, direct evidence of a natural mechanism causing prolonged genomic stasis in the subtype IC lineage was not identified.

### The SARS-CoV-1 escapes in Singapore, Taiwan and China

The initial risk of contracting SARS-CoV-1 through laboratory exposure is very high – even a single mishap could lead to a potential pandemic [58]. This was evidenced by six documented escapes from high-containment virology laboratories: one from a Biological Safety Level (BSL) 3 in Singapore, one from a BSL-4 in Taipei and 4 from the same BSL-3 in Beijing [1]. Despite raising public health alarms, these escapes are not referenced in historical and official reviews of SARS-CoV infections.

#### A laboratory exposure incident in Singapore

In August 2003, a graduate student at the National University of Singapore (NUS) contracted SARS in a BSL-3 laboratory at the Institute of Environmental Health (EHI) Singapore despite handling West Nile Virus (WNV). Examination of the vials revealed that the WNV samples had been cross-contaminated with a SARS-CoV-1 isolate [59]. The student’s sample-preparation techniques were speculated to be the cause of infection. The infected student exposed 8 household contacts, 2 community contacts, 32 hospital contacts and 42 work contacts, of whom 25 were placed under home quarantine [59]. No secondary cases occurred.

Investigations in the laboratory revealed poor record-keeping, missing or defective equipment, a lack of freezers and HEPA/air filter problems, all of which were exacerbated by the student receiving insufficient training in BSL-3 procedures [60].

#### A laboratory spill in Taiwan

In December 2003, research scientist Lieutenant-Colonel (LTC) Chan Jiacong at the Taiwan Military Institute of Preventive Medical Research (IPMR) acquired a SARS-CoV-1 infection, before travelling to Singapore for a conference, where he exposed fellow passengers and airline staff [61].

While working with SARS-CoV-1, LTC Chan found a leaking waste bag; in a hurry to travel, he inadequately disinfected the spill and incorrectly disposed of the waste without appropriate personal protective equipment [62]. WHO investigations revealed numerous safety violations in the laboratory, including poor record-keeping, long work shifts (12–14 h) and the absence of incident-reporting protocol [61]. Original reports cited 95 contacts placed in quarantine, while WHO investigations reported only 74 contacts [1].

#### A laboratory-origin SARS-CoV-1 outbreak in China

**Figure 2.**
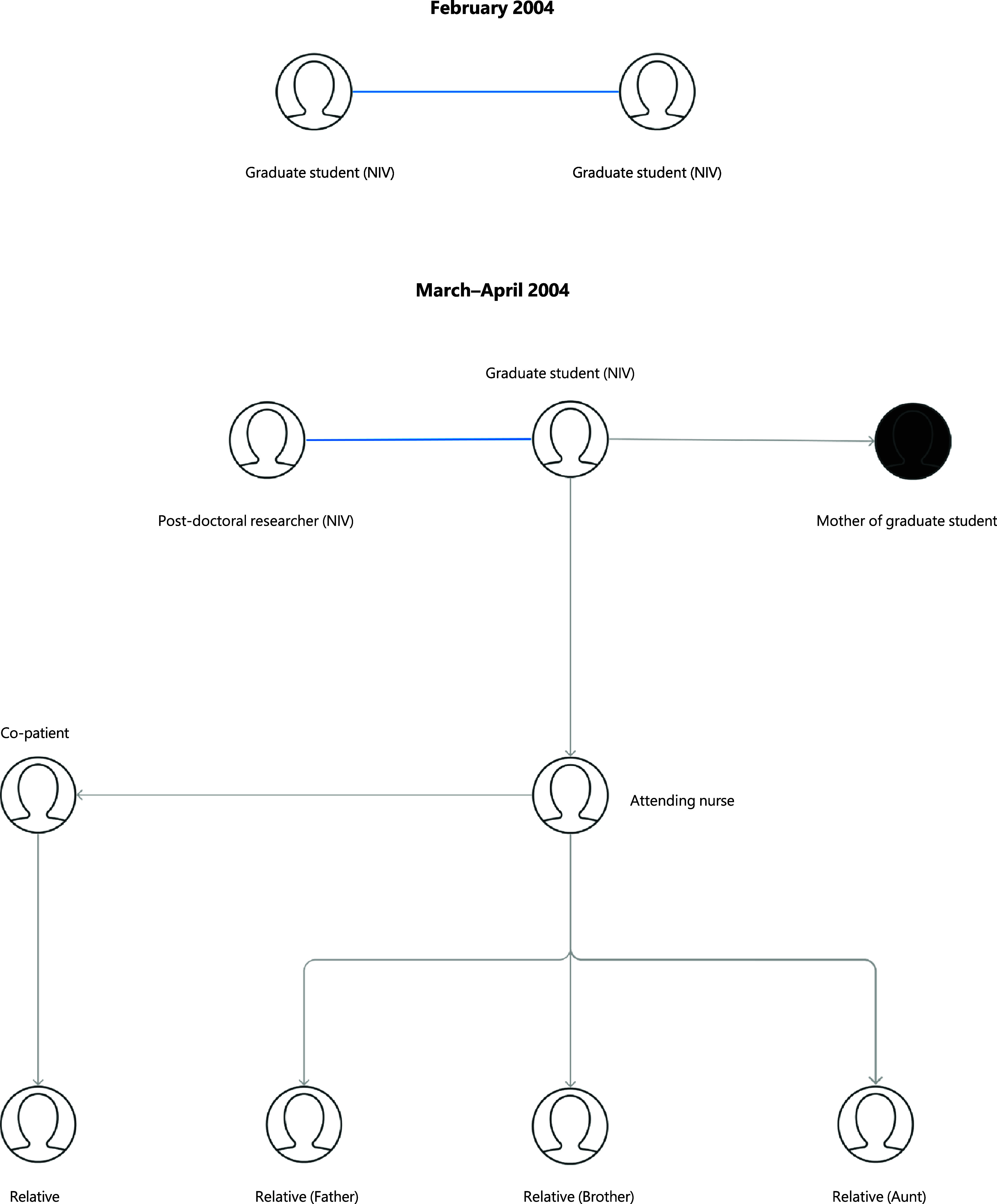
Transmission chains and epidemiological links in laboratory-associated SARS-CoV-1 outbreaks in China (2004). Key: Blue line = independent infection from the same laboratory; Black circle = death.

In April 2004, reports of a nurse with a hospital-acquired SARS-CoV-1 infection came from Beijing, China. She had contracted the illness from a graduate student who was admitted for pneumonia in March. Eventually, the disease spread among their family contacts and healthcare workers over three generations, causing one death [63]. Official reports initially accounted for 9 total cases; however, investigations revealed 2 additional cases from February 2004 [64] (Figure 2).

#### Geographical spread

The graduate student was interning at the viral diarrhoea department of the Chinese National Institute of Virology (NIV) in Beijing, a part of the China Centre for Disease Control (CCDC), and did not work with SARS-CoV nor in a BSL-3 laboratory; the exact mechanism of infection is unknown [1]. She travelled home by train while ill, where her mother developed a severe infection and died as a consequence of attending to her. The nurse who had contracted the illness from the student also transferred it to an additional five individuals (Figure 2) [63]. Investigations found another post-doctoral researcher at NIV who had been infected with SARS-CoV on April 17, 2004 [64]. By the end of April, officially, 747 people were quarantined at NIV [1] and unofficially, over a thousand people [63].

Further investigation found two more graduate students from the same department at NIV who contracted SARS-CoV independently in February [64] (Figure 2). Official reports suggest that one of the doctoral students improperly inactivated a SARS-CoV sample, contaminating the electron microscopy room, from which the second student also acquired the infection [63]. Neither student caused secondary cases, and both recovered. The deactivation solution prepared to inactivate SARS had not been verified or recommended by the Ministry of Health [63].

#### Epidemiological factors

Healthcare workers account for almost 16% of probable SARS-CoV cases with attack rates of >56% [65]. The attack rate (4.23%) and case fatality rate (9.1%) observed in the Beijing escapes were lower than the standard [65], perhaps owing to timely interventions, such as quarantine and isolation, that prevented the outbreak from spreading further. The disease outbreak also occurred in the summer, a low season for virus spread.

#### Timely and accurate reporting/Infodemic

A joint WHO–China report reviewed the cases, although it did not mention two primary cases from February, which were officially discovered in May via IgG testing [64]. As both students were hospitalized in February, the LAIs were known prior to the antigen testing. Perhaps these cases were not disclosed by the institution in the April report [63], as they were later recognised in a WHO report in October 2004 [66]. The WHO highlighted biosafety shortcomings with handling live SARS-CoV and surveillance of LAIs at NIV [1].

### The Foot-and-Mouth virus leak from drainage pipes in Pirbright, England

The United Kingdom was free of Foot-and-Mouth Disease (FMD) for six years until its re-emergence in 2007 [67]. FMD virus (FMDV) predominantly infects cattle, sheep and pigs, with rare cases of mild illness in humans [68]. It is highly transmissible and can spread via contaminated surfaces, aerosols (up to 250 km) and fomites [1, 68]. The FMD outbreak in 2001 incurred a $16 billion loss to the British economy [1]. On 3rd August 2007, the United Kingdom reported an FMD outbreak detected on a cattle farm in Surrey [69]. The pathogen escaped from the Pirbright campus, the only authorized facility in the UK for storing FMDV, specifically the Institute for Animal Health (IAH) and Merial, a vaccine manufacturer.

Initial investigations ruled out aerosol or surface water transmission from Pirbright [70]. Eventually, they revealed a damaged wastewater pipe connecting the Merial vaccine plant to the waste treatment plant in IAH, leaking partially treated waste into the ground and surface water. FMDV-contaminated mud was carried from the campus to the farms via flooding, roads and the tyres of construction vehicles parked at the site [69]. FMDV likely spread further via windborne and fomite transmission and was exacerbated by visitor car parks located near livestock areas [69].

#### Geographical spread

The 2007 FMD outbreak infected 8 premises and 278 animals, necessitating the culling of 1,578 animals and resulting in estimated losses of £200 million [71]. The epidemic comprised two distinct clusters: two farms in the first cluster and six in the second [72]. The intermediary farm between the two infection clusters was missed during initial surveillance [69].

#### Genetic and clinical evidence

The outbreak-causing virus was FMDV serotype O subtype BFS 1860 isolated in 1967, a strain no longer circulating globally [69, 70] but used in large quantities (10,000 l) at the Merial facility and in microquantities at IAH [70]. Both facilities fault the other, leaving the exact location of the outbreak uncertain [1]. Genomic analysis indicated a single escape of FMDV from Pirbright, dating between 13 and 26 July 2007 [71], which caused the August outbreak and re-emerged in mid-September 2007.

The first cases of FMD were discovered in cattle, although pigs are more sensitive to FMDV infection in natural or introduced outbreaks and are often the first animals infected [73]. Additionally, ruminants are more susceptible to airborne infections [74], and the FMDV vaccine production guidelines also focus on efficacy testing and immunization in cattle [75]. The outbreak strain, which shows a greater affinity for ruminants, may indicate a released FMD vaccine strain.

#### Epidemiological factors

The second outbreak cluster began after a significant temporal lag [72]; the epidemic was prematurely deemed over, restrictions on livestock movement were lifted and farm surveillance was eased [69]. Molecular analysis revealed that one of the two originally classified primary farms had been infected by the other, initially overlooked because it lay beyond the 10 km radius for animal epidemic monitoring [76].

Furthermore, risk mapping of the 2007 outbreak indicated an extremely low likelihood of local spread compared to the 2001 FMD outbreak [72], with the sparse livestock density in Surrey raising suspicion of unnatural origins. Moreover, the R_0_ for the 2007 outbreak was ~15, much higher than that for the 2001 outbreak (~4 [71]. While the 2007 strain was more transmissible, there is evidence that a very low level of virus was circulating in infected animals [71].

#### Timely and accurate reporting/Infodemic

The outbreak had a limited infodemic, as animal disease reporting is more strictly enforced and standardized, with delays in reporting risking immediate trade bans and fines. Under the World Organisation for Animal Health (WOAH, formerly known as the OIE) Animal Health Code, member states must notify within 24 h of confirmation [77], while the WHO International Health Regulations (IHR) allow 48 hours [78] with no tangible penalties. Animal health surveillance covers a wider radius with multiple detection points, e.g., mandatory farm reporting, abattoir checks, etc. [79], whereas human health surveillance relies on public health data sharing, often hindered by resource constraints or patient privacy laws [80].

Systemic gaps in communication and reporting were mirrored in site conditions, where long-term damage to drainage pipework was known but unaddressed until FMD emerged in nearby farms [81].

### The Brucella-contaminated waste-gas leak in Lanzhou, China

#### Incident description

The Lanzhou Brucella leak is the largest and longest recorded laboratory-origin outbreak [82], surpassing the 1977 H1N1 and 1979 Sverdlovsk anthrax events [2]. Brucellosis, a globally prevalent zoonotic disease, poses significant public and animal health concerns despite its low human mortality rate [83]. It is transmitted to humans via contact with infected livestock, ingestion of unpasteurized dairy or undercooked meat, and, less commonly, aerosol inhalation, often from laboratory accidents or releases during microbiologic technique [84].

The first cases of *Brucella* spp. infections were detected in November 2019 at the Lanzhou Veterinary Research Institute (LVRI) in Gansu, China, with 181 positive individuals by December [85]. The outbreak originated from the Zhongmu Lanzhou Biopharmaceutical Plant, a state-owned facility producing *Brucella* vaccines for animals [86]. Expired disinfectants led to contaminated waste gas leaking from fermentation tanks downwind to LVRI and neighbouring communities [82].

#### Geographical spread

Although the factory’s manufacturing licence was revoked immediately, transmission continued for at least 12 months [82]. By 30 November 2020, the Health Commission of Lanzhou reported 10,528 cases among 68,571 tested [86]. No deaths were reported, but the full extent of cases is inconclusive due to limited published official data [82, 83].

#### Genetic and clinical evidence

The leaked strain was a *Brucella abortus* A19 vaccine strain, [83]. Owing to its low infectious dose *Brucella* spp. accounts for almost 2% of all LAIs [87], with many documented in China since 1936 [83].

#### Prior incidents

In 2017, a similar outbreak caused by *Brucella suis* S2 vaccines was reported in Gansu, where 51 animal epidemic prevention controllers were positive (attack rate: 24.8%) [85]. In December 2020, another incident occurred at a biological products company in Chongqing, where 61 workers were positive (attack rate: 43.6%). The infection spread to nearby departments [85]. Neither facility complied with biosafety regulations: improper handling techniques, ineffective PPE and ineffective emergency measures were identified [83].

#### Epidemiological factors

*Brucella* was transmitted by aerosols from late July to August 2019 [82, 83], but the attack rate observed with the Lanzhou leak was much lower than typical laboratory exposures (~30%–100%) [88], ranging from ~12.9% to 15.4%. Moreover, no deaths were reported despite a case-fatality rate of 1–2% for brucellosis.

Brucellosis outbreaks in Lanzhou are unusual, with low seroprevalence from 2013 to 2018, and has never been at risk of a *Brucella* epidemic [89]. The sudden increase in cases cannot be attributed to improved surveillance [82], as low levels of brucellosis were detected in high-risk individuals [90].

#### Timely and accurate reporting/Infodemic

The absence of official clinical data from the incident raises concerns about the effectiveness of the response by Chinese authorities [82]. One study revealed that 96 initially exposed individuals were asymptomatic but seroconverted, without mention of the total patients tested or follow-ups [91]. The incident has often been referenced in Chinese publications unrelated to the topic [92, 93]. There were considerable delays in state action, and substandard biosafety and biosecurity regulations remain unresolved [82].

#### Summary of risk factors across laboratory-associated outbreaks

Thematic analysis of the outbreaks identified 19 biological and epidemiological indicators, and 14 institutional, state and social indicators (Table 2). Across the seven outbreaks, the lowest number of indicators (n = 13) was noted in the 2003 SARS escapes, while the highest were observed in the 1955 Cutter Laboratories Polio incident (n = 19) (Supplementary Appendix S2). The number of biological and epidemiological indicators across these outbreaks ranged from 9 to 14, while the institutional state, and social indicators ranged from 3 to 9. The indicators observed across the seven laboratory-associated outbreaks were evaluated to develop risk criteria for flagging possible unnatural origins. The framework consists of five primary criteria, including: unusual strain characteristics, geographical features, epidemiological factors, peculiarities in clinical manifestation and/or affected population(s) and communication to the public and predating biosafety incidents of concern (Table 3).

**Table 2.** Key themes and indicators observed in confirmed laboratory-associated outbreaks and comparative application to SARS-CoV-2

	Outbreak	1955 Polio	1977 H1N1 influenza	1979 Anthrax	1995 VEE	2003 SARS-CoV-1	2007 FMD	2019 Brucella	2019 SARS-CoV-2^†^ (comparative assessment)
	*Biological and epidemiological indicators*								
Strain characteristics	Genetic match to laboratory strain	Vaccine strain	c1950 strain	Military-produced *B. anthracis* strain	c1963 strain	Contaminated WNV sample	c1967 strain	Closely related to RaTG13 (96.1%)	RaTG13 (96.1%)
	Multiple strains found	Subtypes 1–3 found in vaccine lots	Two influenza A subtypes	Four strains circulating					
	Attenuated or engineered strain	Attenuated vaccine strain	Temperature-sensitive	Engineered for bioweapon capacity	Antigenic strain containing live virus			Attenuated vaccine strain	Furin cleavage site
	Unusual virulence		Low		High		Low	Low	High
	Rare form			Inhalational			Aerosol	Inhalational	
	Rare strain in humans	Formaldehyde inactivated strain	Strain no longer circulating in humans		Not circulating for 20 years		Eradicated strain	*B abortus* A19 strain	Novel strain
Geographical features	Unusual size/morphology			Larger aerosol size					
	Emergence in multiple locations	Across western US	China and Soviet Union		Venezuela and Colombia		Multiple farms in nearby regions		Serological evidence in multiple locations
	Emergence in low-risk regions	Idaho			Falcon state / limited equine populations		Spare cattle density also noted	Lanzhou	
	Proximity to laboratory facility	Site where vaccines were administered		Emerged near laboratory	Virology and vaccine laboratories near outbreak epicentre		Primary farms near laboratories	Vaccine and research facilities	Coronavirus research laboratories
Clinical manifestation	Population(s) near facility affected	Schools with vaccine programs		Patients lived/worked near laboratory	Patients lived near laboratory	Patients lived/worked near hospital	Farms near laboratory	Patients lived/worked laboratory	Patients lived/worked near a laboratory
	Unusual clinical signs or pathology	Severe symptoms children / higher rates of paralysi					First signs in cattle		Severe respiratory/neurological complications and chronic symptoms
	Animal deaths / epidemics near site			Yes (wet market animals, dogs in vicinity)	Yes, epizootics (horses, donkeys, mules)	Yes (wet market animals)	Yes (cattle)	Yes (animal outbreaks)	
	Unimmunized population(s) affected	Children and other vaccinee household contacts	Younger people affected (<21)		Children affected largely / unimmunised equines				
Epidemiological spread	Off-peak season	Not during poliovirus season	Not during influenza season			Not during flu season			
	Temporal lag in spread	Cases in waves, lag before secondary transmission	Slow spread in China	Slow spread and long dispersal	Lag between outbreaks		Pause between spread		
	Unusual attack rate	Higher	Varying	Higher	Higher		Higher	Lower	Higher
			A year before	True date after reported start date					Possible low-level circulation by Oct-Nov 2019; remains unproven
		Multiple clusters across US			Clusters across Venezuela and Colombia	Two primary cases discovered retrospectively from antibodies	Two clusters		Multiple early transmission clusters (~5), not a single point source
	*Institutional, state and social indicators*								
Mis/disinformation	Information war on origins	Vaccine misinformation spread	Denial from scientists / officials	Denial from scientists and officials					On-going debate
	Natural origin theories		Virus latency	Contaminated meat from wet market	Virus latency		Illegal movement or imports of animals or animal products		Zoonotic spillover/Cold chain contamination
	Full extent of outbreak not stated		Official reports differ from witness accounts/news	Official reports differ from witness accounts/news	Total number of equine deaths not known			Total number of cases and deaths not published	
	No clinical data released					Cause of exposures not disclosed		No follow-ups on early patient data	Early Wuhan clinical data incompletely shared or delayed
	Irrelevant follow-ups							Studies unrelated to outbreak	
Communication	Delays in government action	Delays in state vaccine agency action		Did not disclose to state officials			6 months	Delays in state action	Delays in international disclosure
	Disclosed to the public at a later date		4–6 months delay in Soviet Union; early 1978 in China	Disclosed years later					Disclosed few months later
	Some cases omitted in official reports			True number of cases and deaths unclear		Two primary cases not mentioned		Lack of official publications	
Biosafety concerns / incidents	Related outbreaks or research studies with implicated pathogens	Pilot IPV studies; Albin strain- contaminated OPV trials	1976 Fort Dix anthrax outbreak		LAIV studies and outbreaks				Coronavirus studies at WIV
	Maintenance and engineering issues			Frequently overlooked, and faulty engineering		Frequently overlooked	Frequently overlooked		Unconfirmed intelligence reports of biocontainment concerns
	Other biosafety issues (inadequate inactivation, disinfection, training, etc.)	Frequent concerns		Multiple regulations overlooked	Difficult to inactivate in the laboratory / vaccine-related outbreaks	Multiple regulations overlooked		Multiple regulations overlooked	Unconfirmed intelligence reports of biosafety inadequacies
	LAI(s) in facility				LAIs via aerosol common	All labs working with live SARS-CoV		LAI outbreak in staff	
	Pathogen release within secondary containment in facility					No SARS-CoV studied in area where student developed infection		LAI outbreak in staff	
	Similar incident(s) in other facilities	Wyeth Laboratories vaccine-associated cases	Research facilities in China and Soviet Union			Singapore, Taiwan incidents		Incidents in vaccine facilities in the region	
	**Total indicators (descriptive, not diagnostic)**	**19**	**17**	**18**	**18**	**13**	**16**	**18**	**19**

*Note:* The listed themes include: strain characteristics (yellow), geographical features (green), clinical manifestation (dark blue), epidemiological factors (blue), mis/disinformation (pink), communication to the public (red) and biosafety concerns/incidents predating the event (purple). ^†^SARS-CoV-2 is included for comparative application of the risk-assessment framework only. Inclusion does not imply confirmation of a laboratory-associated origin.

**Table 3.** Descriptive risk criteria observed in historically documented accidental laboratory-associated outbreaks

Risk criteria	Description
Unusual strain characteristics	Strains that are: Rare Antiquated Eradicated/no longer circulating Novel or emerging, with mutations or signatures of genetic engineering/synthetic biology or adaptation to humans for easy transmission Resistant to prophylactic and therapeutic measures Other factors to consider: The R_0_ (may be higher or lower than their natural counterparts, as an attenuated vaccine strain may be released) Mode of transmission Dose of exposure (dose–response relationship) Increased virulence Unusual environmental stability
Geographical features	Location factors: Emerges in a region for the first time ever Re-emerges after a long period of time Emerges in a region with an otherwise low endemic risk for the implicated pathogen An official laboratory (BSL 2–4) or an unofficial facility engaged in related research is present within a 15-km radius Other factors to consider: Unstable geopolitical environment
Epidemiological factors	Infection dynamics: Sudden emergence of epidemic A point-source, limited outbreak Temporal lags in the spread of disease Atypical temporal context of epidemic, off-peak occurrence Deviations from the natural epidemic pattern Transmission: Deviations from expected modes of transmission, such as inhalational anthrax and inhalational brucellosis Unusually high concentrations in the air, soil and drinking or surface water over a large area, or around the area of emergence of the disease.
Peculiarities in clinical manifestation and affected population(s)	Affected population includes: Laboratory staff and residents residing in the vicinity Naïve persons in otherwise immune populations Clinical signs to consider: Drug or vaccine resistance Unexpected clinical symptoms or pathology (severe disease, complications or chronic disease) Unusually high or low case-fatality rates Increase in patient visits/hospitalisations Sudden unexplainable deaths in animal populations within a 10–25 km radius near the outbreak site
Communication to the public and predating biosafety incidents of concern	Other insights identified prior to the outbreak, during the period of outbreak or post-outbreak: Biosafety concerns or incidents from the institution, or within the region reported prior to the outbreak Delay in reporting the outbreak Mis/disinformation released to the public

*Note:* The listed criteria include: unusual strain characteristics, geographical features and distribution, epidemiological factors and spread, peculiarities in clinical manifestation and affected population(s), communication to the public and predating biosafety incidents of concern.

## Discussion

The study examined recurring epidemiological, operational and governance features that distinguish laboratory-origin outbreaks from natural ones, drawing on historical cases and applying this analytical framework to the origins of SARS-CoV-2 without implying causality. These events are rarely attributable to a single technical failure, but rather an interplay of immediate laboratory-level breaches and systemic deficiencies in governance, oversight and risk communication.

Consistent indicators emerged across the examined outbreaks. Technical failures, such as inadequate handling and transfer of inactivated pathogens and poor maintenance, underpinned the majority of outbreaks. Sudden, unexplained deaths in animal populations within an area have historically served as sentinels for the release of infectious agents and have preceded human case recognition in outbreaks of Anthrax, VEE, Brucella and FMD (Table 2). This reinforces the value of animal surveillance data for outbreak identification, as most pathogens released intentionally or unintentionally are zoonotic.

Epidemiological, spatial and geographical anomalies were recurrent across outbreaks [1], as were atypical strain features [94] (Table 2). Importantly, aside from the Anthrax escape, pathogens were often circulating prior to detection, demonstrating fragmented reporting and disease surveillance systems. Certain outbreaks lacked many biological and epidemiological indicators, but institutional/state/social factors pointed to outbreaks of laboratory origin, i.e., the 2003 SARS-CoV-1 escapes and the 2019 Brucella leak. The opposite was also observed in the 2007 Pirbright FMDV leak, the 1955 Cutter Laboratories Polio incident and the 1995 VEE epidemic, where biological and epidemiological indicators were more abundant than institutional and state factors (Supplementary Appendix S2), suggesting that both classes of indicators should be considered when assessing laboratory origins.

While natural or unnatural origin cannot always be conclusively distinguished, such anomalies provide strong signals of possible unnatural origin [7]. The identified indicators were used to develop a framework of risk criteria for identifying such incidents (Table 3).

Using this framework, the emergence of SARS-CoV-2 exhibited several indicators warranting structured assessment. We emphasise that the presence of these indicators does not establish origin and should not be interpreted independently of virological, epidemiological, and institutional investigations. Initial WHO missions in 2020 and 2021 concluded that a laboratory origin was highly improbable. Subsequent evaluations by the WHO Scientific Advisory Group for the Origins of Novel Pathogens (SAGO) reported that zoonotic spillover remains the most supported hypothesis, however a laboratory-associated incident cannot be excluded due to incomplete access to requested information [95]. The earliest recognized cluster occurred in Wuhan, which hosts two major coronavirus research facilities, including the Wuhan Institute of Virology (WIV), where research on SARS-related coronaviruses had been conducted [96, 97]. Several early cases lacked exposure to the Huanan seafood market [98]. Although environmental sampling detected SARS-CoV-2 contamination at the market, including in wildlife-associated stalls [99], these findings could not distinguish between contamination arising from infected animals and introduction by infected humans. [99]. No intermediate host has been definitively identified to support zoonotic spillover despite extensive sampling. Reports suggesting pre-December 2019 circulation in several countries remain unconfirmed owing to the absence of virus-neutralisation or sequencing data [95]. Clinical anomalies were also present, in particular, unique neuropathological and cardiovascular symptoms were observed in young adults [6, 100]. Moreover, high rates of presymptomatic and asymptomatic transmission were seen in SARS-CoV-2 patients in comparison to the 2002–3 SARS-CoV-1 outbreak, where asymptomatic infection was rare. SARS-CoV-2 achieved effective dissemination due to its widespread asymptomatic carriage in the population leading to undetected community spread.

Genomic features attracting scientific interest include the presence of a furin cleavage site not observed in the closest known sarbecovirus [101], early markers of high virulence [102] and rapid adaptation to human transmission [103]. Although unusual, current analyses demonstrate that these features can arise through natural evolutionary mechanisms. The modified Grunow–Finke tool produced scores consistent with an unintentional laboratory-related event [6]. However, these tools rely on incomplete datasets and assumptions and cannot substitute for direct virological or epidemiological evidence. Consistent with our risk criteria, recurrent themes included unconfirmed biosafety concerns, uncertainties in early transmission dynamics, marked clinical impact, genomic features of interest and multiple early clusters. Although an accidental laboratory origin of SARS CoV-2 has been suggested, no direct evidence supports this scenario, and many experts continue to view natural zoonotic spillover as the more likely pathway. A definitive resolution will require access to the missing data and continued investigation.

All events detailed here were shaped by systemic weaknesses. Fragmented legislation, inadequate oversight and poor governance allowed breaches to go unreported. Unlike animal health systems, international frameworks such as the IHR lack standardized implementation of guidelines for human outbreak reporting. Risk communication failures, whether through delay, omission or disinformation, were a persistent feature undermining public trust and hindering containment efforts.

These findings demonstrate that laboratory-origin outbreaks share a recognizable epidemiological and operational fingerprint, which, paired with systemic governance insights, can strengthen outbreak surveillance. Prevention demands a shift from reliance on technical safeguards to a systems-based approach, with cooperative governance, integrated One Health surveillance, transparent data sharing and proactive risk communication. By embedding biosafety into these broader systems, accidental releases can become rare exceptions rather than recurrent events.

## Supporting information

10.1017/S0950268825100915.sm001 Dhawan et al. supplementary material Dhawan et al. supplementary material

## Data Availability

No primary data was collected for this review. All original source data are available from the cited publications. Data extracted from published studies are presented in the supplementary materials.

## Supplementary material

The supplementary material for this article can be found at http://doi.org/10.1017/S0950268825100915.
