# Supplementary material for: Epidemiological indicators of accidental laboratory-origin outbreaks
Source: Epidemiol Infect. 2026 Jan 2;154:e16. doi: 10.1017/S0950268825100915 (PMC12835948; doi:10.1017/S0950268825100915)
Supplement: Dhawan et al. supplementary material [file S0950268825100915sup001.docx]

# Appendix

Supplementary Table 1. Summary of all accidental laboratory leaks. An accidental laboratory leak was classified as an inadvertent pathogen escape(s) from laboratory settings that breached the secondary containment barrier and was released into the outside environment. Note: Fatality figures reflect officially reported deaths at the time of investigation; where reliable global estimates are unavailable or contested, entries are qualified to indicate uncertainty or under-reporting. “Unknown” indicates that exposure counts could not be reliably reconstructed from available contemporaneous records or retrospective analyses.

| Year | Location | Pathogen | Cases | Exposures | Fatalities | Community cases (Y/N) | Reference |
| --- | --- | --- | --- | --- | --- | --- | --- |
| 1901 | USA | *Clostridium tetani* | Unknown | Unknown | 1 | Yes | [1] |
| 1901 | USA | *Clostridium tetani* | Unknown | Unknown | 9 | Yes | [2] |
| 1902 | India | *Yersinia pestis* | Unknown | Unknown | 19 | Yes | [3] |
| 1928 | Australia | *Staphylococcus aureus* | 21 | Unknown | 12 | Yes | [4] |
| 1931 | United Kingdom | *Microsporum gypseum* | 4 | Unknown | 0 | Yes | [5] |
| 1932 | Germany | *Mycobacterium tuberculosis* | 228 | 23 | 72 | Yes | [6] |
| 1947 | South Africa | *Yersinia pestis* | 2 | 0 | 1 | Yes | [7] |
| 1948 | USA | *Coccidioides immitis* | 3 | Unknown | 0 | Yes | [8] |
| 1948 | Germany | *Coxiella burnetii* | 23 | Unknown | 0 | Yes | [9] |
| 1949 | USA | *Coccidioides immitis* | 15 | 19 | 0 | Yes | [8] |
| 1955 | USA | Poliovirus | 40000 | Unknown | 10 | Yes | [10, 11] |
| 1956 | USA | *Histoplasma capsulatum* | 1 | Unknown | 0 | Yes | [12] |
| 1966 | United Kingdom | Variola minor | 73 | Unknown | 0 | Yes | [13] |
| 1967 | Germany | Marburg | 31 | Unknown | 7 | Yes | [14] |
| 1971 | USA | *Coxiella burnetii* | 15 | 5 | 0 | Yes | [15] |
| 1971 | Russia | Variola major | 10 | Unknown | 3 | Yes | [16] |
| 1972 | United Kingdom | Variola major | 4 | Unknown | 2 | Yes | [13] |
| 1976 | South Korea | Hantavirus | 9 | 1 | 0 | Yes | [17] |
| 1976 | USA | Influenza virus H1N1 | 532 | Unknown | 25 | Yes | [18] |
| 1977 | China/Soviet Union | Influenza virus H1N1 | Unknown | Unknown | Not reliably quantified | Yes | [13] |
| 1978 | USA | Foot-and-Mouth Disease Virus | Unknown | Unknown | Unknown | Yes | [19] |
| 1978 | USA | Foot-and-Mouth Disease Virus | 2 | 0 | Unknown | Yes | [19] |
| 1978 | United Kingdom | Variola major | 2 | Unknown | 1 | Yes | [13] |
| 1979 | Russia | *Bacillus anthracis* | 96 | Unknown | 105 | Yes | [13] |
| 1980 | USA | *Coxiella burnetii* | 65 | 72 | 0 | Yes | [20] |
| 1980 | USA | Foot-and-Mouth Disease Virus | 9 | Unknown | Unknown | Yes | [19] |
| 1980 | Venezuela | Variola major | 1 | Unknown | 0 | Yes | [21] |
| 1981 | USA | Foot-and-Mouth Disease Virus | 4 | Unknown | Unknown | Yes | [19] |
| 1982 | Spain | *Brucella melitensis* | 28 | 136 | 0 | Yes | [22] |
| 1982 | Belgium | *Brucella melitensis* | 2 | Unknown | 0 | Yes | [23] |
| 1982 | Canada | *Coxiella burnetii* | 59 | 272 | 0 | No | [24] |
| 1987 | USA | Foot-and-Mouth Disease Virus | 1 | Unknown | Unknown | Yes | [19] |
| 1987 | USA | Herpes virus B | 4 | 0 | 1 | Yes | [25] |
| 1987 | Australia | Avian paramyxovirus-1 | 1 | 0 | 0 | No | [26] |
| 1988 | USA | *Brucella abortus* | 5 | Unknown | 0 | Yes | [27] |
| 1988 | USA | *Leptospira interrogans* | 2 | 0 | 0 | Yes | [28] |
| 1989 | United Kingdom | *Staphylococcus* spp. | 1 | 0 | 0 | Yes | [29] |
| 1992 | The Netherlands | Poliovirus | 1 | 1 | 0 | Yes | [30] |
| 1995 | Venezuela/Colombia | Venezuelan equine encephalitis virus | ~100,000 | Unknown | ~300 | Yes | [13] |
| 1998 | USA | Rotavirus | 9 | Unknown | 0 | Yes | [31] |
| 1999 | Russia | Crimean Congo Hemorrhagic Fever Virus | 69 | Unknown | 6 | Yes | [19] |
| 2000 | India | Poliovirus | 3 | Unknown | 0 | No | [32] |
| 2000 | Russia | Vaccinia | 8 | Unknown | 0 | Yes | [33] |
| 2001 | China | Hantavirus | Unknown | Unknown | Unknown | No | [34] |
| 2002 | India | Poliovirus | 7 | Unknown | 0 | Yes | [32] |
| 2003 | Taiwan | SARS-CoV-1 | 1 | 74 | 0 | Yes | [35] |
| 2003 | USA | West Nile Virus | 0 | 50 | 0 | Yes | [36] |
| 2004 | USA | Foot-and-Mouth Disease virus | 6 | Unknown | 0 | Yes | [37] |
| 2004 | China | Hantavirus | Unknown | Unknown | Unknown | No | [38] |
| 2004 | China | SARS-CoV-1 | 11 | 747 | 1 | Yes | [35] |
| 2005 | USA | *Bacillus anthracis* | 0 | 2 | 0 | Yes | [19] |
| 2007 | USA | *Brucella abortus* | Unknown | 196 | 0 | No | [39] |
| 2007 | United Kingdom | Foot-and-Mouth Disease virus | 278 | Unknown | 1578 | Yes | [40] |
| 2007 | Belgium | *Shigella sonnei* | 5 | Unknown | 0 | Yes | [41] |
| 2008 | USA | *Brucella* spp. | 1 | Unknown | Unknown | Yes | [42] |
| 2009 | USA | Titi Monkey Adenovirus | 2 | 20 | 0 | Yes | [43] |
| 2012 | United Kingdom | *Bacillus anthracis* | 0 | 2 | 0 | No | [44] |
| 2014 | USA | Influenza A virus H5N1 | 0 | Unknown | 0 | No | [45] |
| 2014 | Belgium | Poliovirus | Unknown | Unknown | 0 | No | [46] |
| 2014 | China | Poliovirus | 1 | Unknown | 0 | Yes | [47] |
| 2015 | USA | Bacillus anthracis | Unknown | Unknown | 0 | No | [48] |
| 2017 | Netherlands | Poliovirus | 1 | 1 | 0 | No | [49] |
| 2018 | Samoa | Measles | 2 | 0 | 2 | Yes | [50] |
| 2018 | USA | NS | Unknown | 55 | 0 | No | [51, 52] |
| 2019 | China | *Brucella* *abortus* A19 | 10,528 | Unknown | 0 | Yes | [53] |
| 2019 | USA | NS | NS | Unknown | 0 | No | [51, 54] |
| 2019 | USA | NS | 1 | NS | 0 | No | [51, 55] |
| 2021 | Taiwan | SARS-CoV-2 | 1 | 110 | 0 | No | [56] |
| 2022 | Netherlands | Poliovirus | 1 | 50 | 0 | Yes | [57] |
| 2024 | USA | Rabies | Unknown | 15 | 0 | Yes | [58] |

*NS = not stated

Supplementary Table 2. Summary of risk indicators across the outbreaks. The total number of biological, epidemiological, institutional, state, and social indicators is outlined for each outbreak. This table demonstrates the comparative features as described in the literature for context only. We do not draw inferences on origin from these comparisons.

| Outbreak | Biological and epidemiological indicators | Institutional, state, and social indicators | Total indicators |
| --- | --- | --- | --- |
| 1955 Poliovirus | 14 | 5 | 19 |
| 1977 Influenza A virus H5N1 | 11 | 6 | 17 |
| 1979 Anthrax | 10 | 8 | 18 |
| 1995 Venezuelan equine encephalitis virus | 13 | 5 | 18 |
| 2003-4 SARS-CoV-1 | 5 | 8 | 13 |
| 2007 Foot-and-Mouth Disease virus | 13 | 3 | 16 |
| 2019 *Brucella abortus* A19 | 9 | 9 | 18 |
| 2019 SARS-CoV-2 | 11 | 8 | 19 |

**References**

1. DeHovitz, R.E., *The 1901 St Louis Incident: The First Modern Medical Disaster.* Pediatrics, 2014. **133**(6): p. 964-965.

2. Lilienfeld, D.E., *The first pharmacoepidemiologic investigations: national drug safety policy in the United States, 1901-1902.* Perspect Biol Med, 2008. **51**(2): p. 188-98.

3. Ross, R., *The Inoculation Accident at Mulkowal.* Nature, 1907. **75**(1951): p. 486-487.

4. Hooker, C., *Diphtheria, Immunisation and the Bundaberg Tragedy: A Study of Public Health in Australia.* Health and History, 2000. **2**.

5. Parish, H.J. and S. Craddock, *A Ringworm Epizootic in Mice.* Br J Exp Pathol, 1931. **12**(4): p. 209-12.

6. Fox, G.J., M. Orlova, and E. Schurr, *Tuberculosis in Newborns: The Lessons of the "Lübeck Disaster" (1929-1933).* PLoS Pathog, 2016. **12**(1): p. e1005271.

7. Burmeister, R.W., W.D. Tigertt, and E.L. Overholt, *Laboratory-acquired pneumonic plague. Report of a case and review of previous cases.* Ann Intern Med, 1962. **56**: p. 789-800.

8. Everett Hanel Jr., R.H.K., *Laboratory-acquired mycoses*, F.D. Department of the Army, Editor. 1967: Frederick, Maryland.

9. Pike, R.M., *Laboratory-associated infections: incidence, fatalities, causes, and prevention.* Annu Rev Microbiol, 1979. **33**: p. 41-66.

10. Nathanson, N. and A.D. Langmuir, *The Cutter Incident. Poliomyelitis Following Formaldehyde- Inactivated Poliovirus Vaccination in the United States during the Spring of 1955. Ii. Relationship of Poliomyelitis to Cutter Vaccine.* Am J Hyg, 1963. **78**: p. 29-60.

11. Offit, P.A., *The Cutter incident, 50 years later.* N Engl J Med, 2005. **352**(14): p. 1411-2.

12. Spicknall, C.G., R.W. Ryan, and A. Cain, *Laboratory-acquired histoplasmosis.* N Engl J Med, 1956. **254**(5): p. 210-4.

13. Furmanski, M., *Escaped Viruses*, in *Laboratory Escapes and “Self-fulfilling prophecy” Epidemics*, S.s.W.G.o.C.a.B. Weapons, Editor. 2014, Center for Arms Control and Nonproliferation: Washington DC.

14. Pavlin, B.I., *Calculation of incubation period and serial interval from multiple outbreaks of Marburg virus disease.* BMC Res Notes, 2014. **7**: p. 906.

15. Curet, L.B. and J.C. Paust, *Transmission of Q fever from experimental sheep to laboratory personnel.* Am J Obstet Gynecol, 1972. **114**(4): p. 566-8.

16. Broad, W.J.M., Judith, *Traces of Terror: The Bioterror Threat | Report Provides New Details Of Soviet Smallpox Accident*, in *The New York Times*. 2002.

17. Lee, H.W. and K.M. Johnson, *Laboratory-acquired infections with Hantaan virus, the etiologic agent of Korean hemorrhagic fever.* J Infect Dis, 1982. **146**(5): p. 645-51.

18. Neustadt, R.E. and H.V. Fineberg, in *The Swine Flu Affair: Decision-Making on a Slippery Disease*. 1978, National Academies Press (US)

Copyright © National Academy of Sciences.: Washington (DC).

19. Manheim, D. and G. Lewis, *High-risk human-caused pathogen exposure events from 1975-2016.* F1000Res, 2021. **10**: p. 752.

20. Meiklejohn, G., et al., *Cryptic epidemic of Q fever in a medical school.* J Infect Dis, 1981. **144**(2): p. 107-13.

21. Pan American Health, O., *ACCIDENTAL SMALLPOX VACCINATION IN VENEZUELA.* PAHO. Epidemiological Bulletin;1(5),1980, 1980.

22. Ollé-Goig, J.E. and J. Canela-Soler, *An outbreak of Brucella melitensis infection by airborne transmission among laboratory workers.* Am J Public Health, 1987. **77**(3): p. 335-8.

23. Goossens, H., et al., *Brucella melitensis: person-to-person transmission?* Lancet, 1983. **1**(8327): p. 773.

24. Simor, A.E., et al., *Q fever: hazard from sheep used in research.* Can Med Assoc J, 1984. **130**(8): p. 1013-6.

25. *B-virus infection in humans--Pensacola, Florida.* MMWR Morb Mortal Wkly Rep, 1987. **36**(19): p. 289-90, 295-6.

26. Morgan, C., *Import of animal viruses opposed after accident at laboratory.* Nature, 1987. **328**(6125): p. 8-8.

27. Braymen, D.T. *Conditions Contributing to a Brucella Exposure to Laboratory Personnel*. in *32nd American Association for Biosafety and Biosafety (ABSA) conference*. 1989. USA.

28. Bolin, C.A. and P. Koellner, *Human-to-human transmission of Leptospira interrogans by milk.* J Infect Dis, 1988. **158**(1): p. 246-7.

29. Grist, N.R. and J.A. Emslie, *Infections in British clinical laboratories, 1988-1989.* J Clin Pathol, 1991. **44**(8): p. 667-9.

30. Mulders, M.N., et al., *Genetic Analysis of Wild-Type Poliovirus Importation into The Netherlands (1979–1995).* The Journal of Infectious Diseases, 1997. **176**(3): p. 617-624.

31. Murphy, T.V., et al., *Intussusception among infants given an oral rotavirus vaccine.* N Engl J Med, 2001. **344**(8): p. 564-72.

32. Deshpande, J.M., S.S. Nadkarni, and Z.A. Siddiqui, *Detection of MEF-1 laboratory reference strain of poliovirus type 2 in children with poliomyelitis in India in 2002 & 2003.* Indian J Med Res, 2003. **118**: p. 217-23.

33. World Health Organization, W., *2000 - Accidental exposure to smallpox vaccine in the Russian Federation*, A.C.o.V.V. Research, Editor. 2000, WER.

34. Hu LL, Z.Y., Wang WH., *Epidemiological investigation of hemorrhagic fever in population caused by laboratory animals.* Chinese Journal of Epidemiology, 2001(23): p. 223-4.

35. Normile, D., *Infectious diseases. Mounting lab accidents raise SARS fears.* Science, 2004. **304**(5671): p. 659-61.

36. *Unattributed: FedEx Package With Dead Virus Explodes.*, in *Huron Daily Tribune*. 2003.

37. USGA, *High-Containment Biosafety Laboratories: DHS Lacks Evidence to Conclude That Foot-and-Mouth Disease Research Can Be Done Safely on the U.S. Mainland*. 2004.

38. Zhang HL, D.X., Zhang YZ, et al, *The investigation of epidemic hemorrhagic fever in Yunnan province.* China Journal of Zoonoses, 2004(20): p. 44-5.

39. Noe, R., et al., *Update: Potential exposures to attenuated vaccine strain Brucella abortus RB51 during a laboratory proficiency test - United States and Canada, 2007.* JAMA The Journal of the American Medical Association, 2008. **299**: p. 891-893.

40. DEFRA, *Foot and Mouth Disease confirmed in cattle, in Surrey*. 2007.

41. Schrijver, K., et al., *Een laboratoriuminfectie met Shigella sonnei gevolgd door een cluster van secundaire infecties.* Tijdschrift Voor Geneeskunde, 2007. **63**: p. 686-690.

42. Alison Young, N.P., *Inside America's secretive biolabs*, in *USA Today*. 2015.

43. Chen, E.C., et al., *Cross-species transmission of a novel adenovirus associated with a fulminant pneumonia outbreak in a new world monkey colony.* PLoS Pathog, 2011. **7**(7): p. e1002155.

44. Sample, I., *Revealed: 100 safety breaches at UK labs handling potentially deadly diseases*, in *The Guardian*. 2014.

45. Centers for Disease Control and Prevention, C., *Report on the inadvertent cross-contamination and shipment of a laboratory specimen with influenza virus H5N1*. 2014.

46. Duizer, E., et al., *Risk assessment, risk management and risk-based monitoring following a reported accidental release of poliovirus in Belgium, September to November 2014.* Euro Surveill, 2016. **21**(11): p. 30169.

47. Chesnais, M., et al., *Opening a 60-year time capsule: sequences of historical poliovirus cold variants shed a new light on a contemporary strain.* Virus Evol, 2024. **10**(1): p. veae063.

48. Centers for Disease Control and Prevention, C., *Report on the Potential Exposure to Anthrax*. 2014.

49. Bandyopadhyay, A.S., et al., *Facility-Associated Release of Polioviruses into Communities-Risks for the Posteradication Era.* Emerg Infect Dis, 2019. **25**(7): p. 1363-1369.

50. Craig, A.T., A.E. Heywood, and H. Worth, *Measles epidemic in Samoa and other Pacific islands.* The Lancet Infectious Diseases, 2020. **20**(3): p. 273-275.

51. Young, A., *Pandora's Gamble: Lab Leaks, Pandemics, and a World at Risk*. 2023: Hachette Nashville.

52. Advisory, F.D.M., *Flooding to Fort Detrick Steam Sterilization Plant*, F.D.P.A. Office, Editor. 2018: Maryland.

53. zhu, x., et al., *Laboratory-Acquired Brucella Infection and S2 Vaccine infection Events in China*. 2020.

54. (name redacted), D.D.f.P., *USAMRIID Biosafety Lapses*, n.r. Letter to USAMRIID commander, Editor. 2019, Public Health Service and Implementation Science: CDC Division of Select Agents and Toxins.

55. Jorgensen, S., e.t.F.C.a.M.s.h. offcials, Editor. 2019, Federal Select Agent Responsible Official: USAMRIID.

56. Everington, K., *Taiwan Lists 3 Possible Ways COVID Lab Infection Occurred*, in *Taiwan News*. 2021.

57. Duizer, E., et al., *Wild poliovirus type 3 (WPV3)-shedding event following detection in environmental surveillance of poliovirus essential facilities, the Netherlands, November 2022 to January 2023.* Euro Surveill, 2023. **28**(5).

58. Leigh Searcy, M.E., *Plumbing leak causes possible rabies exposures at Frankfort state lab.*, in *LEX 18*. 2024, WLEX Lex18 News: Lexington, Kentucky.
